# Heterogeneity in the association between weather and pain severity among patients with chronic pain: a Bayesian multilevel regression analysis

**DOI:** 10.1097/PR9.0000000000000963

**Published:** 2022-01-12

**Authors:** Belay B. Yimer, David M. Schultz, Anna L. Beukenhorst, Mark Lunt, Huai L. Pisaniello, Thomas House, Jamie C. Sergeant, John McBeth, William G. Dixon

**Affiliations:** a Centre for Epidemiology Versus Arthritis, University of Manchester, Manchester, United Kingdom; b NIHR Greater Manchester Biomedical Research Centre, Manchester Academic Health Science Centre, University of Manchester, Manchester, United Kingdom; c Department of Earth and Environmental Sciences, Centre for Atmospheric Science, University of Manchester, Manchester, United Kingdom; d Centre for Crisis Studies and Mitigation, University of Manchester, Manchester, United Kingdom; e Department of Biostatistics, Harvard T. H. Chan School of Public Health, Boston, MA, USA; f Discipline of Medicine, The University of Adelaide, Adelaide, Australia; g School of Mathematics, The University of Manchester, Manchester, United Kingdom; h Centre for Biostatistics, University of Manchester, Manchester, United Kingdom

**Keywords:** Chronic pain, Weather, Musculoskeletal diseases, Multilevel modelling, Observational studies

## Abstract

**Introduction:**

Previous studies on the association between weather and pain severity among patients with chronic pain have produced mixed results. In part, this inconsistency may be due to differences in individual pain responses to the weather.

**Methods:**

To test the hypothesis that there might be subgroups of participants with different pain responses to different weather conditions, we examined data from a longitudinal smartphone-based study, Cloudy with a Chance of Pain, conducted between January 2016 and April 2017. The study recruited more than 13,000 participants and recorded daily pain severity on a 5-point scale (range: no pain to very severe pain) along with hourly local weather data for up to 15 months. We used a Bayesian multilevel model to examine the weather–pain association.

**Results:**

We found 1 in 10 patients with chronic pain were sensitive to the temperature, 1 in 25 to relative humidity, 1 in 50 to pressure, and 3 in 100 to wind speed, after adjusting for age, sex, belief in the weather–pain association, mood, and activity level. The direction of the weather–pain association differed between people. Although participants seem to be differentially sensitive to weather conditions, there is no definite indication that participants' underlying pain conditions play a role in weather sensitivity.

**Conclusion:**

This study demonstrated that weather sensitivity among patients with chronic pain is more apparent in some subgroups of participants. In addition, among those sensitive to the weather, the direction of the weather–pain association can differ.

## Abstract 2

Supplemental Digital Content is Available in the Text.

Weather sensitivity among patients with chronic pain is a phenomenon more apparent in some participant subgroups.

## 1. Introduction

There is a strong belief among patients with chronic pain that pain severity is influenced by the weather.^17,23^ However, studies investigating the association between weather and pain have yielded conflicting results.^2,21^ One possibility for this lack of consensus is that some people within the population are highly sensitive to the weather, others are less sensitive, and some are not sensitive.^16,21,23^ Such differences among individuals, including the subjective and highly personal nature of pain experience, are well known.^11^ These individual differences are not merely a byproduct of idiosyncrasies in the reporting of pain but may be a result of interindividual differences in cerebral activation evoked by the same painful stimulus.^6,9^ However, most of the previous studies have focused on the average effect of weather on pain severity at a population level and have not investigated individual differences.^8,25^ Understanding individual variation and the factors contributing to individual differences in pain may provide insights into pain mechanisms. However, assessing individual variation requires fitting models explicitly designed to account for individual-specific responses and their associated uncertainty intervals. Without repeated observations of the same individuals over a sufficient time, this is not possible. Two studies attempted to model weather–pain association at an individual level using a multilevel modelling framework that explicitly models individual-level heterogeneity.^3,10^ However, the small sample size and limited follow-up hampered the robustness of their analysis. As a result, there is no robust evidence for heterogeneity in the weather–pain association.

Recently, we conducted a large UK-based smartphone study, Cloudy with a Chance of Pain (www.cloudywithachanceofpain.com), recruiting more than 13,000 patients across the UK over 15 months.^7^ Participants with a range of underlying pain conditions tracked their daily symptoms through the study smartphone application (app) for 6 months or more while the GPS in the smartphone enabled local weather data collection. An analysis of the Cloudy with a Chance of Pain data set by Dixon et al.^7^ demonstrated higher relative humidity and wind speed and lower atmospheric pressure were associated with increased pain severity. The analysis used a case-crossover method to generate population-level estimation of the weather–pain association that corrected for the individual difference in unmeasured baseline factors. However, the Cloudy with a Chance of Pain data set also provided a unique opportunity for exploring individual-level heterogeneity. In this study, we test the hypothesis that there is an association between the weather and pain severity that is only apparent in a subgroup of participants. We then examine the extent to which the difference in underlying pain condition captures individual heterogeneity.

## 2. Methods

### 2.1. Design and study sample

Cloudy with a Chance of Pain (www.cloudywithachanceofpain.com)^7^ was conducted between January 20, 2016, and April 20, 2017, to understand the relationship between weather and pain. A total of 13,207 users across the UK over the 12-month recruitment period downloaded the study smartphone application. Information including age, sex, underlying pain condition, and participants' belief about the weather–pain association (“How likely do you think it is that the weather is associated with pain?” measured on a 1- to 10-point scale with 1 being “not at all likely” and 10 being “extremely likely”) was recorded at baseline. Participants were requested to submit their pain severity level on an ordinal scale with 5 categories and 9 other variables, including mood, activity, and fatigue, daily. Participants were followed up from their first pain severity level entry up to the last pain severity level entry. A total of 10,584 participants had completed baseline information and at least one pain entry, with 6850 (65%) participants remaining in the study beyond their first week.^7^ A detailed description of the study is presented in the research conducted by Dixon et al.^7^ Ethical approval was obtained from the University of Manchester Research Ethics Committee (ref: ethics/15522) and from the NHS IRAS (ref: 23/NW/0716).

### 2.2. Inclusion criteria

Participants were included in the final cohort for this analysis if they fulfilled the following criteria: (1) downloaded the app, (2) provided consent, (3) completed the baseline questionnaire, and (4) contributed at least 2 days of pain severity data.

### 2.3. Primary outcome measure

The outcome of interest was the daily self-reported pain severity level recorded on a 1- to 5-point ordinal scale (1: no pain, 2: mild pain, 3: moderate pain, 4: severe pain, and 5: very severe pain). Participants were asked to report self-reported pain severity level every day using the smartphone application, prompted by a daily notification at 6:24 pm.

### 2.4. Exposures

The exposures of interest in this study were 4 state weather parameters, namely the average daily temperature, pressure, relative humidity, and wind speed a participant was exposed to each day. Study participants' locations were recorded at each hour of the day using the study app. Weather information, including temperature, pressure, relative humidity, and wind speed, was retrieved by linking participants' locations to the nearest Met Office weather station. When participants were outside the UK during the study period, their data were not analysed because we were unable to link to non-UK weather stations.

### 2.5. Covariates

We considered age (in years), sex, baseline beliefs about the association between weather and pain, the daily record of mood (on a 1- to 5-point scale; 1: depressed, 2: feeling low, 3: not very happy, 4: quite happy, and 5: very happy) and exercise (on a 1- to 5-point scale; 1: no exercise, 2: less than 30 minutes of light activity, 3: 30+ minutes of light activity, 4: less than 30 minutes of strenuous activity, and 5: 30+ minutes of strenuous activity) as possible factors^7^ that may influence the weather–pain association.

### 2.6. Statistical analysis

The association between weather and pain severity was tested with a multilevel ordinal probit model.^14,18,24^ This model was considered ideal for this analysis because it allowed an estimate of the average response across the group through the fixed-effect terms, and it could explicitly model participant-level heterogeneity using random-effect terms. The model allows every participant in our study to have their unique response to the weather while also improving population-average estimates by pooling information across participants.^13^ The model also appropriately handles the primary outcome's ordinal scale and irregular (ie, unbalanced) repeated measurements.^20^

We developed a multivariable multilevel model that included the 4 state weather parameters adjusted for age, sex, belief, time since entry to the study, mood, and exercise as fixed effects. A linear relationship was assumed for all variables in the model except for time since entry to the study, which was modelled nonparametrically using a cubic spline^26^ (ie, the data entirely determined the relationship between time and the response). We assumed a linear relationship between weather parameters and pain severity because the complex nonparametric relationship did not produce a better fit. We included time since entry to the study in the model as a means of filtering out unmeasured time-varying factors that may influence a participant's pain severity reports. In addition to the abovementioned fixed-effect terms, the model included 5 correlated participant-specific random effects, namely a random intercept and a random effect for each of the 4 state weather parameters. The random intercept term captures the between-participant variation not explained by the baseline factors. The random effects for the weather parameters allow the weather effects to vary over study participants.

The Bayesian estimation approach assuming noninformative priors,^15^ described in detail in Section 2 of the supplementary file (available at http://links.lww.com/PR9/A145), was followed to estimate model parameters. We examined the trace plots and the posterior distribution plot and performed posterior predictive checks^12^ to assess model convergence. We reported the estimated regression coefficients ($\hat{\text{β}}$) along with their associated 95% credible intervals. The regression coefficients represent the change in the *z* score or probit index for a one-unit change in the weather parameter. To quantify the change in the predicted probability for each pain severity response level for a one-unit change in the weather parameter, we used a summary measure called marginal effect at the mean.^1^ The marginal effect at the mean represents the marginal effect of the explanatory variables of interest while holding the other variables in the model at their respective mean values. Furthermore, we presented the participant-level regression coefficients with a 95% credible interval as a forest plot along with the population-level effects ($\hat{\text{β}}$). We divided participants into groups that are statistically distinct from one another by determining whether their credible intervals overlap.^19^ The prevalence of the participant's underlying diagnosis was then compared between groups, with the data presented as a bar plot. Owing to statistical power issue, we were unable to perform a statistical test on the difference in weather sensitivity by the participant's underlying diagnosis. We used the R package brms^4^ based on Stan^5^ to fit the model. The R source code is made available at belayb/Cloudy-Probit: Bayesian Multilevel analysis (github.com).

## 3. Results

### 3.1. Participants' characteristics

Of the 13, 000 participants recruited for the study, a total of 6213 participants who had completed baseline information, submitted at least 2 days of pain reports, and had hourly location data sufficient to retrieve complete weather information to produce daily means were included in the analysis. Study participants included in the analysis had a mean age of 49 years (SD: 13.0); most of them were female individuals (82%), and most of the participants believed in an association between weather and their pain (median score 7 of 10, interquartile range [IQR]: 6–9) (Table 1). Approximately 35% of the participants experienced unspecified arthritis, followed by osteoarthritis (29%) and fibromyalgia (27%) (Table 1). The characteristics of those included in the analysis were similar to the full cohort of participants (Table S1 in the supplementary file, available at http://links.lww.com/PR9/A145). The participants included in the analysis were followed up for a median of 106 days (IQR: 53–215). On average, they contributed pain severity data for 65% of the days during the period when they were actively contributing data to the study. Overall, participants tended to report mild or moderate pain (2 or 3 on our 5-point scale) approximately 70% of the time (Figure S1 in the supplementary file, available at http://links.lww.com/PR9/A145).

**Table 1:** Baseline characteristics of study participants.

Characteristics	Final cohort (N = 6213)
Demographics	
Female, N (%)	5519 (82.4)
Age, mean (SD)	48.68 (13.0)
Diagnosis, N (%)*	
Arthritis (type not specified)	2135 (34.4)
Osteoarthritis	1797 (28.9)
Fibromyalgia/chronic widespread pain	1707 (27.5)
Rheumatoid arthritis	1176 (18.9)
Neuropathic pain	975 (15.7)
Chronic headache (including migraine)	630 (10.1)
Ankylosing spondylitis/spondyloarthropathy	552 (8.9)
Gout	213 (3.4)
Other/no medical diagnosis	1179 (19.0)
Belief in weather–pain association	
Belief that the weather influences pain on a scale of 1–10, median (IQR)	7 (6–9)

* Participants may report more than one pain condition, and when they do, they are counted multiple times in the abovementioned table.

IQR, interquartile range.

### 3.2. Population-level weather–pain association

Table 2 summarizes parameter estimates and their associated 95% credible intervals for the Bayesian multilevel ordinal probit model. At a population level, participants exposed to high relative humidity (0.041, 95% CI: 0.034–0.048) or high wind speed (0.012, 95% CI: 0.009–0.014) have a higher likelihood of experiencing a higher level of pain (Table 2). Similarly, participants exposed to low temperatures (−0.003, 95% CI: −0.005 to −0.001) or low pressures (−0.010, 95% CI: −0.015 to −0.005) have a higher likelihood of experiencing a higher level of pain. That is, an increase in relative humidity by 10 percentage points increases the probability of reporting moderate pain or above by 1.5%, and an increase in wind speed by 1 m·s^−1^ increases the probability of reporting moderate pain or above by 0.40%. Similarly, an increase in temperature by 1°C decreases the probability of reporting moderate pain or above by 0.1%. An increase in pressure by 10 mbar decreases the probability of reporting moderate pain or above by 0.4% (Table 2). In general, the population level estimated that weather–pain association for all considered weather parameters were modest.

**Table 2:** Association between weather and pain—parameter estimates from the Bayesian multilevel ordinal probit model.

Weather parameters	Estimate ($\hat{\text{β}}$)*	95% credible interval	Marginal effects at mean (MEM)†
Temperature (per 1°C)	−0.003	(–0.005 to −0.001)	−0.001
Pressure (per 10 mbar)	−0.010	(–0.015 to −0.005)	−0.004
Relative humidity (per 10%)	0.041	(0.034 to 0.048)	0.015
Wind speed (per 1 m·s^–1^)	0.012	(0.009 to 0.014)	0.004

* The model is adjusted for age (in years), sex, belief, mood, and exercise.

† MEM represents the change in probability of experiencing moderate pain or above as the weather parameter value increases.

### 3.3. Exposure effect heterogeneity

**Figure 1.**
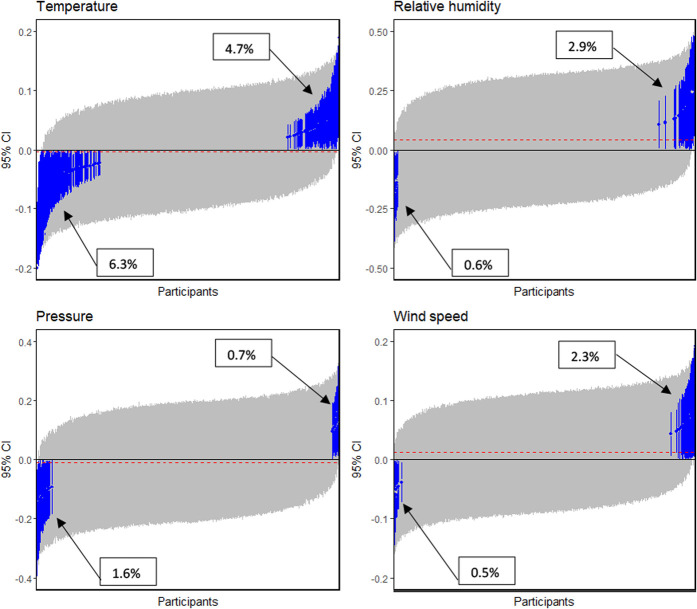
Heterogeneity in weather–pain association. The 95% credible interval for each of the 4 estimated weather effects for each participant sorted by their median values of the estimated effect sizes. Effect sizes are on the latent scale. Intervals shown in blue do not cross zero. The horizontal dotted red line is the population average weather effect on pain severity, consistent with the population-level result listed in Table 2.

We evaluated the participant-level weather–pain associations to identify subgroups within the population who were sensitive to the weather. Figure 1 shows the estimated participant-level regression coefficients and their associated credible intervals for each weather parameter ranked by their median values. We divided participants into groups that are statistically distinct from one another by determining whether their credible intervals overlap. For each of the considered weather parameters, this method identifies 2 distinct clusters (both coloured blue) and a third cluster (coloured grey) for the participants who cannot be statistically distinguished from the members of the 2 distinct clusters. These 3 clusters are given names based on the direction of the weather–pain association: low-value sensitive (ie, participants with negative posterior credible intervals), high-value sensitive (ie, participants with positive posterior credible intervals), and undetermined (ie, participants with credible intervals that overlap with zero). In this study, we considered the name not sensitive instead but settled on undetermined because lack of statistical significance does not mean the absence of a relationship.

Most of the participants belonged to the undetermined cluster for all weather parameters, implying that, for most of the participants, there is not enough evidence to indicate that they possess sensitivity to the weather–pain association. The size of the low-value sensitive and high-value sensitive clusters varies by weather parameter (Figure 1). For example, there were a similar proportion of participants for whom relatively lower temperature was associated with a higher level of pain (6.3%) as there were participants (4.7%) for whom the higher temperature was associated with an increase in their pain, resulting in a very modest overall effect of temperature. On the other hand, the participant-level regression coefficients of relative humidity and wind speed were skewed to the right of zero. More participants (2.9% for relative humidity and 2.2% for wind speed) were sensitive to higher values of these weather parameters than to lower values (0.6% for relative humidity and 0.6% for wind speed). Similarly, proportionally more participants (1.6%) were sensitive to low pressure than high pressure (0.7%). Most of the participants (72.5%) classified as weather sensitive possessed sensitivity to a single weather parameter (Figure S3 in the supplementary material, available at http://links.lww.com/PR9/A145).

**Figure 2.**
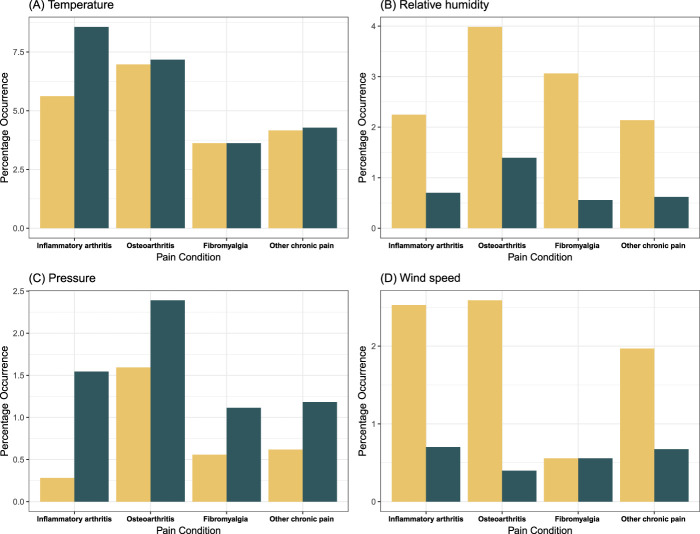
Distribution of weather sensitivity group by underlying pain conditions of participants. Rheumatoid arthritis and ankylosing spondylitis or spondyloarthropathy are grouped as inflammatory arthritic pain. Yellow bars represent high-value sensitive clusters, and green bars represent low-value sensitive clusters.

To understand the role of the participant's underlying disease diagnosis on weather sensitivity, we explored the distribution of the weather sensitivity group in each of the pain conditions of participants. Figure 2 presents the distribution of low-value sensitive and high-value sensitive clusters by participant's disease diagnosis for each of the 4 weather parameters. To simplify the analysis, we grouped various diseases into one of the 4 categories: osteoarthritis, fibromyalgia or chronic widespread pain, inflammatory arthritic pain (rheumatoid arthritis and ankylosing spondylitis or spondyloarthropathy), and other chronic pain. For clarity, we considered only participants with a single diagnosis (n = 3355) in Figure 2. Based on visual inspection of Figure 2, there are for the most part no major differences in the prevalence of weather sensitivity observed between participant's disease diagnosis, although there is perhaps a hint that participants with inflammatory arthritis are more commonly sensitive to low temperatures and have a greater differential sensitivity to pressure.

## 4. Discussion

### 4.1. Summary of principal findings

This study tested the hypothesis that there is an association between the weather and pain severity that is only apparent in a subgroup of participants using a large longitudinal data set. The data presented in this study support that hypothesis. After adjusting for age, sex, belief in the weather–pain association, mood, and activity level for each of the 4 weather parameters considered (ie, the average daily temperature, pressure, relative humidity, and wind speed a participant was exposed to each day), we identified 3 statistically distinct clusters of patients with chronic pain who were each influenced by the weather differently: low-value sensitive, high-value sensitive, and undetermined. Eleven percent of participants were sensitive to temperature, of which 6.3% were sensitive to low temperature. On the other hand, most of those sensitive to relative humidity and wind speed were high-value sensitive (2.9% of 3.5% and 2.3% of 2.8%, respectively). Similarly, most of those sensitive to pressure were low-value sensitive (1.6% of 2.3%).

This study also examined the role of the underlying conditions (ie, participant's disease diagnosis) on their sensitivity to the weather. There is no definite indication of individual underlying pain conditions explaining individual-specific weather–pain association, although participants with inflammatory arthritis may have been more sensitive to cold than the other conditions.

### 4.2. Methodological strengths

This study sets individual variation at the forefront and aims to quantify the influence of weather on pain severity at the individual level. Previous studies focus on an average effect of weather on pain severity at a population level.^8,25^ In the presence of individual heterogeneity, the weak weather–pain association at a population level based on this approach does not rule out the possibility of a stronger weather–pain association at an individual level. Indeed, the lack of a population-level association might be because the study participants are composed of about an equal number of participants affected by the weather in opposite directions, thereby cancelling out the effect in the population as a whole. In the case of a statistically significant positive or negative association at the population level, and in the presence of heterogeneity in the weather–pain relationship, it is problematic to use the resulting population estimates to provide clinical advice to an individual patient because the population-level estimates may not meaningfully apply to individuals. This issue underscores the importance of explicitly modelling individual-level heterogeneity. However, without repeated observations of the same individuals over a long period, it is impossible to quantify individual-level heterogeneity and identify subgroups that behave differently.

This study uses a large data set obtained from the Cloudy with a Chance of Pain study,^7^ which produced a unique data set by recruiting more than 13,000 participants with sustained daily self-reported data and accurate weather information to which they were exposed over many months. The data set also recorded daily self-reported mood and activity level, which were ideal for estimating the weather effect that was acting not through these variables. We used a multilevel modelling approach to analyse the data and investigate the influence of weather on pain severity. The modelling approach allows every participant in the study to have their unique response to the weather while also improving the population-level average estimate by pooling information across study participants.^20^ When estimating the average effect of weather on the population, the multilevel modelling approach prevented oversampled individuals from unfairly dominating the result by considering the differential uncertainty across participants.^20^

A limitation in our study was that our study participants were aware of the study objective, which may raise possible information bias where observed weather could influence participants' symptom reporting. However, our analysis has been adjusted for previous belief, and hence, information bias will not fully explain the observed association. Also, the findings from this study cannot necessarily be extrapolated to different climates where the weather is different.

### 4.3. Comparison with other studies

Previous studies have reported a relatively higher percentage of weather-sensitive individuals. For example, Fagerlund et al.^10^ investigated individual differences in weather sensitivity using a multilevel modelling framework. They found significant individual differences, with a subgroup of patients (20%) behaving contrarily to most patients by reporting increased pain with increased atmospheric pressure. Similarly, Bossema et al.^3^ used a multilevel modelling approach to investigate individual heterogeneity. They reported a positive association between the weather variables (ie, temperature, sunshine duration, perception, pressure, and relative humidity) and pain in approximately one‐third of the patients, a negative association in one‐third of the patients, and no association in the remaining patients. The 2 studies considered only patients with fibromyalgia. Compared with our result, the higher reported percentage may be attributed to the difference in the methodology. For example, Bossema et al.^3^ used Pearson correlation between the fibromyalgia symptom and the weather condition for each patient to identify individual-level association rather than using the multilevel model used to estimate population effect in their analysis.

Two studies^16,22^ among others^2^ examined subgroups sensitive to the weather by analysing each participant's data individually and reported substantial difference among individuals in weather sensitivity. However, such analysis is prone to overfitting and may lead to spurious associations.

### 4.4. Implications of the study

This study demonstrated that weather sensitivity among patients with chronic pain is a phenomenon more apparent in some subgroups of participants. In addition, among those sensitive to the weather, the direction of the weather–pain association can differ. When considering future potential benefits and applications of understanding the association between weather and pain, such as developing a “pain forecast” to help patients predict their forthcoming pain, our results would support the need for a personalised prediction.

## Disclosures

W.G. Dixon has received consultancy fees from Google and AbbVie unrelated to this study.

## Appendix A. Supplemental digital content

Supplemental digital content associated with this article can be found online at http://links.lww.com/PR9/A145.

## Supplementary Material

SUPPLEMENTARY MATERIAL
