# Supplementary material for: Heterogeneity in the association between weather and pain severity among patients with chronic pain: a Bayesian multilevel regression analysis
Source: Pain Rep. 2022 Jan 12;7(1):e963. doi: 10.1097/PR9.0000000000000963 (PMC8759613; doi:10.1097/PR9.0000000000000963)
Supplement: SUPPLEMENTARY MATERIAL [file painreports-7-e963-s001.pdf]

Supplementary Material for "Heterogeneity in the association between weather and pain severity among patients with chronic pain: a Bayesian multilevel regression analysis"

Belay B. Yimer[1,2,*], David M. Schultz[3,4], Anna L. Beukenhorst[5], Mark Lunt[1,2], Huai Leng Pisaniello[1,6], Thomas House[7], Jamie C. Sergeant[1,8], John McBeth[1,2], William G. Dixon[1,2]

[1]Centre for Epidemiology Versus Arthritis, University of Manchester, Manchester, United Kingdom

[2]NIHR Greater Manchester Biomedical Research Centre, Manchester Academic Health Science Centre, University of Manchester, Manchester, United Kingdom[3]Centre for Atmospheric Science, Department of Earth and Environmental Sciences, University of Manchester, Manchester, United Kingdom

[4]Centre for Crisis Studies and Mitigation, University of Manchester, Manchester, United Kingdom

[5]Department of Biostatistics, Harvard T. H. Chan School of Public Health, Boston, Massachusetts, United States

[6]Discipline of Medicine, The University of Adelaide, Adelaide, Australia

[7]School of Mathematics, The University of Manchester, Manchester, United Kingdom

[8]Centre for Biostatistics, University of Manchester, Manchester, United Kingdom

1. Introduction

This supplementary file presents further details and additional materials not presented in the main manuscript.

2. Further detail on the Multilevel model

2.1 Model formulation

The model was formulated as follows. Let Y_{ij} denote the jth pain-severity level report for the ith participant at time t_{ij} and x_{ij} denote the accompanying vector of covariate values at the time t_{ij}, $i = 1, \dots, N$, and $j = 1, \dots, n_i$. We assume that the ordinal response Y_{ij} with $K = 5$ ordered categories (or levels) can be viewed as a censored observation from a hidden continuous variable, Y_{ij}^* ,

$$Y_{ij} = y_{ij} \leftrightarrow c_{k-1} < Y_{ij}^* \le c_k, \qquad Y_{ij} \in \{1, \dots, K\},$$

where $-\infty \equiv c_0 < c_1 < \cdots < c_K \equiv \infty$ are suitable threshold parameters [1]. That is, a response for the ith individual at time t_{ij} occurs in pain-severity category k ($Y_{ij} = k$) if the latent response process Y_{ij}^* exceeds the threshold value c_{k-1}, but does not exceed the threshold value c_k.

Then, for the specification of the relationship between the unobserved Y_{ij}^* and the vector of regressors $\boldsymbol{x}_{ij}$, we follow a mixed-effect model-type specification [1]

$$Y_{ij}^* = \boldsymbol{x}_{ij}^T \boldsymbol{\beta} + \boldsymbol{Z}_{ij} \boldsymbol{u}_i + \epsilon_{ij},$$

where $\boldsymbol{\beta}$ are population-level regression coefficients, $\boldsymbol{u_i} = (u_{1i}, \dots, u_{Li})$ are patient-specific, normally distributed, $u_{li} \sim N(0, \sigma_{\mathrm{u_l}}^2)$ random effects describing the heterogeneity (i.e., individuals' deviation from the population-level effect) among different individuals, $\boldsymbol{x}_{ij}$ is $N \times p$ design matrix for the fixed effect, $\boldsymbol{Z}_{ij}$ is a $N \times L$ design matrix corresponding to the random-effect vectors $\boldsymbol{u}_i$, and ϵ_{ij} is the underlying error, where p is the number of variables included in the fixed effect, including the global intercept, and L is the number of random components, including the random intercept. We assume a normal distribution for ϵ_{ij} leading to a probit model. Also, we assume independence between ϵ_{ij} and $\boldsymbol{u}_i$ (i.e., a homogeneous residual variance conditional on the fixed effect and random effect).

2.2 Estimation

We used the Markov-chain Monte Carlo (MCMC) simulations to fit the above multilevel probit model. Bayesian estimation requires prior information for each of the model parameters. We assumed a weakly informative but proper prior for all model parameters. That is, we assumed a normal *N*(0, 2.5) prior for each of the regression coefficients ($\boldsymbol{\beta}$) and a half-Student-*t* prior with a mean of zero, three degrees of freedom, and a scale parameter of 10 [2] for the hyperparameters ($\sigma_{u_l}^2, l = 1, \dots, L$). All models were fitted using the R package *brms* [3] based on Stan [4] using four chains of 8000 iterations each, thinned to every 10 trials where the first

4000 iterations are considered as burn-in trials. A Gelman–Rubin diagnostic ($\hat{R}$) [2] was used to confirm model convergence.

2.3 Model goodness of fit

We used a posterior predictive check approach to evaluate the fitted models' goodness of fit [5]. The posterior predictive check works by comparing the observed data to the simulated data from the fitted model. To generate the data used for posterior predictive checks (PPCs), we simulate from the posterior predictive distribution, which is the distribution of the outcome variable implied by a model after using the observed data to update our beliefs about unknown model parameters. If a model is a good fit for the data, then the simulated data should look like the observed data.

3. Additional results

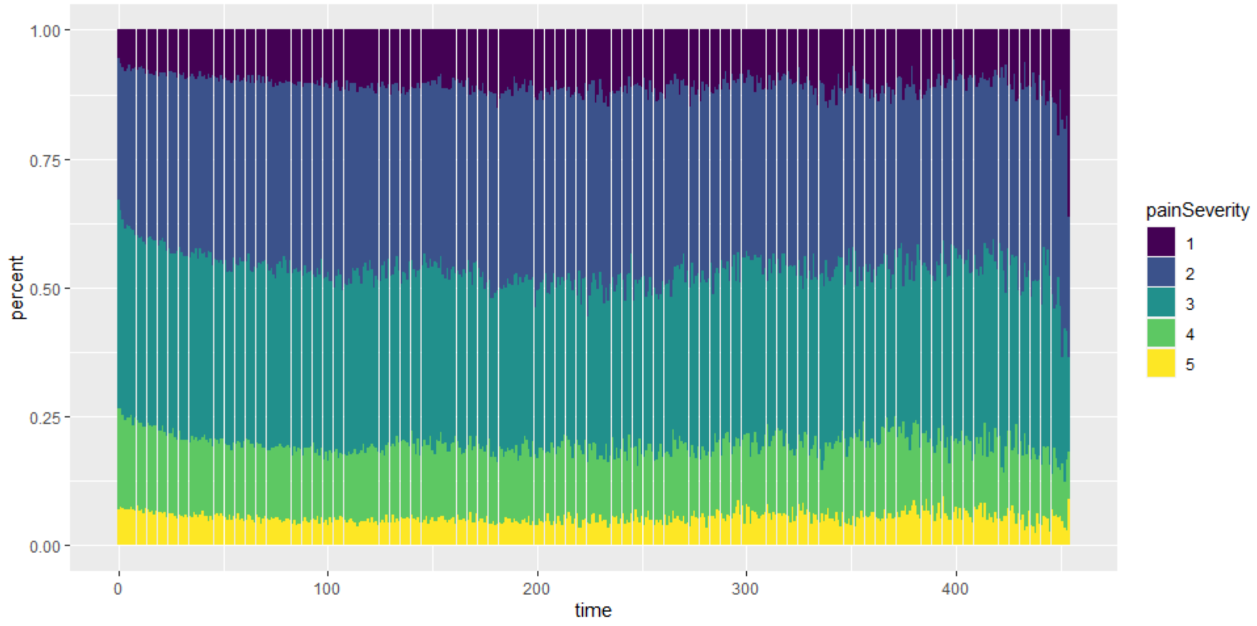

Figure S1: The observed proportion of pain severity response over time.

Table S1. Baseline characteristics of study participant included in the analysis along with the full cohort.

Characteristics	Final cohort (N = 6213)	Full cohort (N = 10584)[+]
**DEMOGRAPHICS**		
Female: N (%)	5519 (82.4)	8554 (80.8)
Age: mean (sd)	48.68 (13.0)	47.87 (13.2)
**DIAGNOSIS: N (%)** [*]		
Arthritis (type not specified)	2135 (34.4)	3662 (34.6)
Osteoarthritis	1797 (28.9)	2552 (24.1)
Fibromyalgia/chronic widespread pain	1707 (27.5)	2791 (26.4)
Rheumatoid arthritis	1176 (18.9)	1954 (18.5)
Neuropathic pain	975 (15.7)	1593 (15.1)
Chronic headache (including migraine)	630 (10.1)	1085 (10.3)
Ankylosing spondylitis/ spondyloarthropathy	552 (8.9)	923 (8.7)
Gout	213 (3.4)	371 (3.5)
Other/no medical diagnosis	1179 (19.0)	2758 (26.1)
**BELIEFS IN WEATHER–PAIN ASSOCIATION:**		
Belief that the weather influences pain on a scale of 1–10: median (IQR)	7 (6–9)	7 (6–9)

** Participants may report more than one pain condition, and when they do, they are counted multiple times in the above table.*

+ Only particpants that had reponded to the baseline questionare included in the full cohort.

Table S2. Estimated variance component parameters from the multilevel model

**Heterogeneity Measures**		
The standard error for random intercept ($\boldsymbol{\sigma_{Int}}$)	10.582	(9.975, 11.202)
The standard error for random temperature effect ($\boldsymbol{\sigma_{T}}$)	0.051	(0.049, 0.053)
The standard error for random pressure effect ($\boldsymbol{\sigma_{P}}$)	0.101	(0.095, 0.107)
The standard error for random relative humidity effect ($\boldsymbol{\sigma_{RH}}$)	0.138	(0.130, 0.146)
The standard error for random wind speed effect ($\boldsymbol{\sigma_{WS}}$)	0.051	(0.047, 0.054)

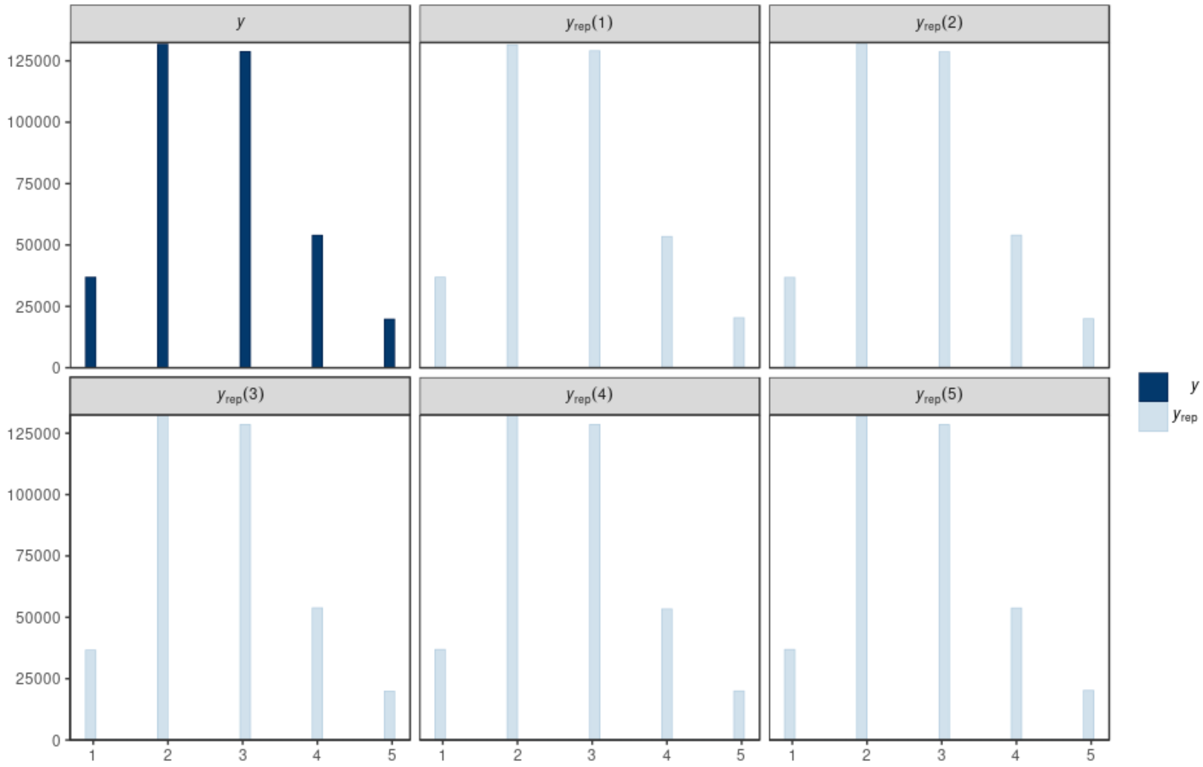

Figure S2: Posterior predictive checks: y is the observed data and $y_{rep}(1) - y_{rep}(5)$ *are simulated data from the final model.*

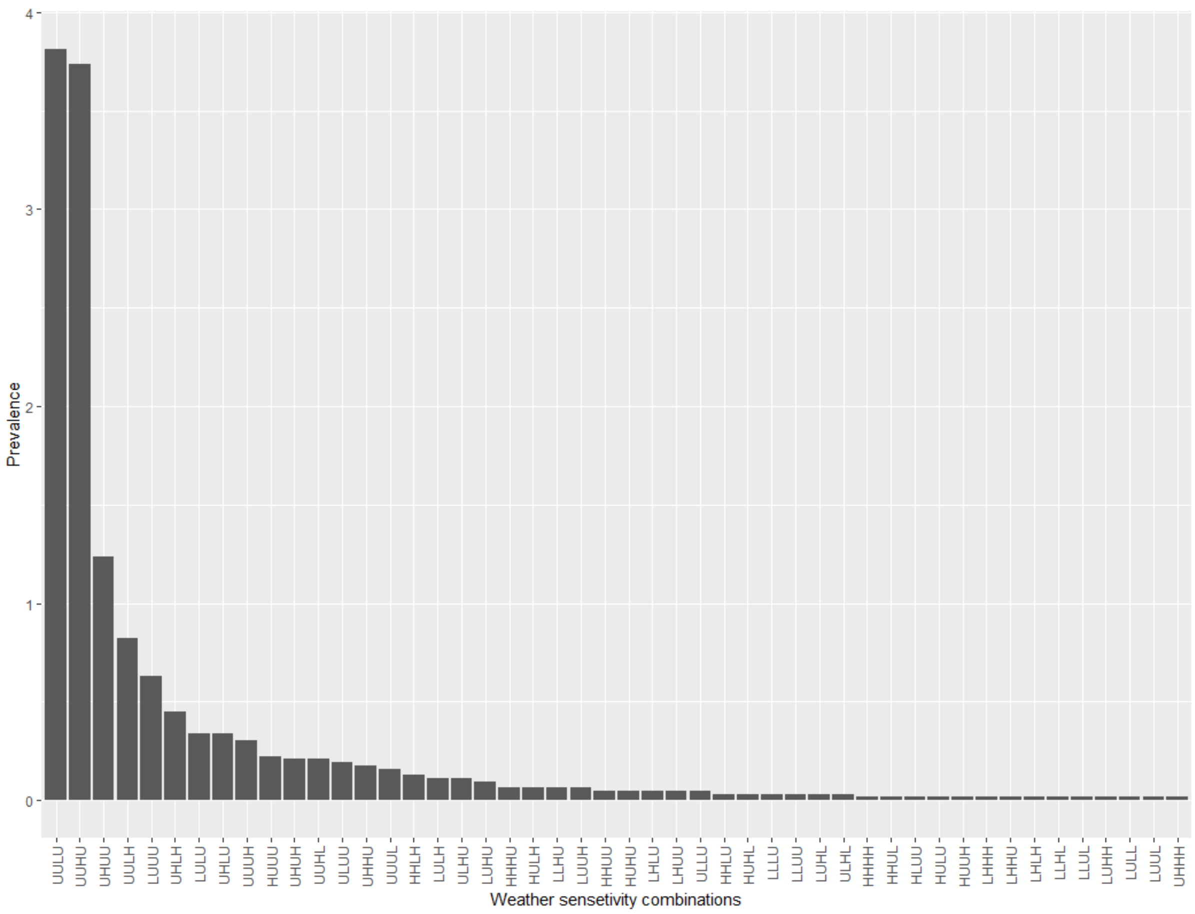

Figure S3. The percentage of individuals sensitive to different weather parameters. U denotes undetermined, L denotes low value-sensitive, and H denotes high-value sensitive. The combinations is in Pressure-Humidity-Temperature-Windspeed order. For example, UULU denotes the percentage of individuals with undetermined pressure effect, undetermined humidity effect, sensitive to low temperature, and undetermined windspeed effect.

References

1. Varin, C. and C. Czado, *A mixed autoregressive probit model for ordinal longitudinal data.* Biostatistics, 2009. **11**(1): p. 127-138.
2. Gelman, A. and J. Hill, *Data analysis using regression and multilevel/hierarchical models*. 2006: Cambridge university press.
3. Bürkner, P.-C., *brms: An R package for Bayesian multilevel models using Stan.* Journal of Statistical Software, 2017. **80**(1): p. 1-28.
4. Carpenter, B., et al., *Stan: A probabilistic programming language.* Journal of statistical software, 2017. **76**(1).

5. Gabry, J., et al., *Visualization in Bayesian workflow.* Journal of the Royal Statistical Society: Series A (Statistics in Society), 2019. **182**(2): p. 389-402.
